# Leveraging multigenerational health data to enhance mental disorder risk prediction: a population-based cohort study

**DOI:** 10.1186/s12888-025-07323-z

**Published:** 2025-09-25

**Authors:** Amani F. Hamad, Barret A. Monchka, James M. Bolton, Leslie L. Roos, Mohamed Elgendi, Lisa M. Lix

**Affiliations:** 1 Department of Community Health Sciences, Rady Faculty of Health Sciences, University of Manitoba, 753 McDermot Avenue, Winnipeg, MB R3E 0T6 Canada; 2 George and Fay Yee Centre for Healthcare Innovation, University of Manitoba, Winnipeg, MB Canada; 3 Department of Psychiatry, Rady Faculty of Health Sciences, University of Manitoba, Winnipeg, MB Canada; 4 Department of Biomedical Engineering and Biotechnology, Khalifa University, Abu Dhabi, United Arab Emirates; 5 Centre for Biotechnology, Khalifa University, Abu Dhabi, United Arab Emirates; 6 Department of Health Sciences and Technology, ETH Zurich, Zurich, Switzerland

**Keywords:** Mental disorders, Comorbidities, Prediction, Family history, Multigenerational linkage, Administrative data, Cohort study

## Abstract

**Background:**

Mental disorders are highly prevalent, and comorbidities between physical and mental health conditions are common. Physical comorbidities and family health histories may improve the accuracy of mental disorder risk prediction. We developed prediction models for mental disorder risk using comprehensive individual and family mental and physical health histories.

**Methods:**

We conducted a population-based cohort study using administrative Health data in Manitoba, Canada, and included adults between 1977 and 2020 with linkages to at least one parent and one grandparent. Mental disorders (mood and anxiety, substance use and psychotic disorders) for individuals, parents and grandparents were identified in inpatient and outpatient Health records. Predictors included demographics, family history of mental disorders and 130 health conditions in individuals, parents and grandparents. We used the Least Absolute Shrinkage and Selection Operator (LASSO) logistic regression to build prediction models that sequentially included health conditions in individuals, parents and grandparents. Predictive performance was evaluated using the area under the receiver operating characteristic curve (AUC), sensitivity, specificity, positive predictive value, negative predictive value and Brier score.

**Results:**

Of 125 070 individuals identified, 109 359 had no preexisting mental disorder. 52.9% were males and 52.8% were urban residents. 39 651 (36.3%) had a recorded diagnosis of mental disorders during follow-up. Predictive models incorporating Health histories of individuals, parents, and grandparents achieved the best predictive performance. Amongst all mental disorders, psychotic and substance use disorders had the highest AUCs of 0.78 (95% confidence interval (CI) 0.75–0.81) and 0.75 (95% CI 0.73–0.76), respectively. Key predictors included comorbid mental disorders, gastrointestinal conditions, female infertility and family history of dementia, gastrointestinal and metabolic conditions.

**Conclusions:**

Individual and family histories of physical and mental conditions improved mental disorder risk prediction, though accuracy was only moderate, highlighting the need for further refinement of risk prediction.

**Supplementary Information:**

The online version contains supplementary material available at 10.1186/s12888-025-07323-z.

## Background

Mental disorders are prevalent conditions; worldwide, 970 million people, which is equivalent to one in every eight people, are living with a mental disorder [1]. In the US, 23% of adults had a mental illness in 2021, including 6% with a serious mental illness that substantially interfered with a major life activity [2]. More than 18% of Canadians aged 15 and older had a mood, anxiety or substance use disorder in 2022 [3]. The burden of mental disorders is significant, as they influence relationships, employment, education and physical health [4–9]. 

Mental and physical health conditions often co-occur [8, 10–15]. For example, up to 41% of individuals with physical illnesses, including cancer, stroke and multiple sclerosis, have a major depressive disorder, which is significantly higher than the 4.7% prevalence in the general population [16–20]. The relationship between mental and physical health conditions can be attributed to shared biological, psychosocial and environmental pathways [21]. For instance, individuals with cancer are more likely to develop depression and anxiety in response to diagnosis, relapse and survivorship [21]. Shared biological pathways, including dysfunctional immune responses, are also implicated in the comorbidity of cancer and mental disorders [21]. Additionally, social determinants of health, including socioeconomic status, are associated with both mental and physical health conditions, making social disadvantage a risk factor for mental and physical illness comorbidity [22–27]. 

The co-occurrence of physical and mental health conditions may extend across generations due to shared genetics and environmental exposures. For example, chronic inflammation and immune dysregulation can be passed down or influenced by early life exposures, which impact mental health across generations [28–32]. Additionally, health behaviours and stressors often cluster within families, leading to similar patterns in physical and mental health conditions. Therefore, family history, particularly of physical conditions that overlap via biological or behavioural mechanisms with mental health, may offer predictive value for mental disorder risk in offspring. An association between parental physical health conditions, such as autoimmune diseases, diabetes, and obesity, and offspring mental disorder risk has also been observed [33–36]. Therefore, considering physical health comorbidities and family history of diseases can potentially improve the accuracy of incident mental disorder risk prediction.

Identifying individuals at high risk of developing a mental disorder is essential for risk stratification, prevention and early intervention, resource allocation and health system planning. Existing risk prediction models of mental disorders relied on small samples, disease-specific populations, self-reported health status, and a limited number of predictors and did not consider histories of physical health conditions [37–46]. In addition, prior studies that examined family history have typically focused only on parents and have not considered grandparent health histories. As a result, these models may not capture the full range of individual and familial factors that influence mental health risk. This represents a critical knowledge gap, particularly given our knowledge about physical and mental health comorbidity within and across generations. Addressing this knowledge gap is important for improving early identification and intervention strategies. To our knowledge, no previous studies have developed a mental disorder risk prediction model using a broad set of objectively measured physical and mental health conditions across three generations in a general population cohort. Therefore, we aimed to develop mental disorder risk prediction models using population-based, objectively identified multigenerational mental and physical health histories to assess their contribution to model performance and to identify potential novel familial predictors.

## Methods

### Study design and data sources

This was a retrospective population-based cohort study using administrative healthcare data in Manitoba, Canada, between April 1^st^ 1974 and March 31 st 2020. The data are contained in the Manitoba Population Research Data Repository (Repository) housed at the Manitoba Centre for Health Policy, which includes healthcare encounters for nearly the entire Manitoba population due to the universal and publicly funded healthcare system [47]. Individual records can be linked across the different datasets using Personal Health Identification Numbers. Five datasets were used: the Manitoba Health Insurance Registry, Hospital Abstracts, Medical Claims, the Hospital Newborn to Mother Link Registry and Canada Census.

The Manitoba Health Insurance Registry is a longitudinal population-based registry that includes individual-level demographic, residential and family composition information through Family Identification Numbers (FRNs) since 1970. The Registry was used to create the study cohort. Hospital Abstracts consist of hospital records since 1970 and include demographic, diagnosis (up to 25 diagnosis codes) and procedure (up to 20 procedure codes) information. Medical Claims contain records of physician visits in offices and outpatient departments since 1970 and include diagnosis and service codes. Three revisions of the International Classification of Diseases (ICD) System have been used over time to report diagnoses: the eighth (ICD-8), the ninth with clinical modifications (ICD-9-CM) and the tenth with Canadian adaptations (ICD-10-CA, in Hospital Abstracts only) (see Additional file 1). Hospital Abstracts and Medical Claims were used to ascertain individual and family members’ diagnoses of mental and physical conditions. The Hospital Newborn to Mother Link Registry links mother’s delivery and newborn’s birth hospital records and was used to verify and supplement linkages to the mothers in the cohort. Canada Census is a nationwide population survey conducted by Statistics Canada every five years. It was used to obtain income quintiles, an area-level income measure [48]. 

### Population

The cohort included individuals in the Registry who were 18 years or older from April 1 st 1977 to March 31 st 2020 with linkages to at least one parent and one grandparent. The individuals were required to have at least three years of continuous health insurance enrollment prior to and directly following index date; index date was April 1st of the year individuals turned 18 years or April 1 st 1977, whichever occurred later. The individuals were also required to have a parent and a grandparent with at least three years of continuous health insurance enrollment prior to index date. Linkages to parents and grandparents were created using FRNs in the Registry, which are assigned to individuals and adults in the same household. Mothers generally share the same FRN with their children. In contrast, fathers only share an FRN with the mother and child if the parental relationship is formally reported to the provincial health agency. FRNs change over time when children become adults or family structure changes. Both historical and current FRNs are recorded in the Registry, allowing individuals and families to be followed longitudinally despite these changes. Individuals with a mental disorder diagnosis prior to index date were excluded. Individuals were followed up until the outcome occurred, migration out of the province, death or end of study period (see Additional file 2).

### Outcome measures

Study outcomes included incident diagnoses of 1) any mental disorder, 2) mood and anxiety disorders, 3) substance use disorders and 4) psychotic disorders, identified in Hospital Abstracts and Medical Claims using algorithms adopted from previous literature [49]. Additional file 3 lists the ICD codes used to identify study outcome measures. Any mental disorder (omnibus definition) was defined as having any mood and anxiety, substance use or psychotic disorders. Mood and anxiety disorders were defined as one or more hospital records or two or more outpatient physician contacts within three years with a relevant ICD code. Substance use and psychotic disorders were defined as one or more hospital records or outpatient physician contacts with a relevant ICD code.

### Predictors

The predictors included demographic information at index date of sex (male, female), income quintile (Q1 – lowest to Q5 – highest), region of residence (urban, rural) and family history of the outcome in each of parents and grandparents. Additionally, 130 mental and physical health conditions in individuals (excluding the outcome of interest), either parent or any grandparent, were included. The health conditions were based on an adaptation of the Clinical Classification Software, which provides crosswalks from multiple ICD versions into mutually exclusive and clinically meaningful categories of health conditions [50, 51]. Health conditions were identified in Hospital Abstracts and Medical Claims from 1974 until the end of follow-up (see Additional file 2). They were defined as one or more hospital records or outpatient physician contacts with a relevant ICD code [51]. 

### Statistical analysis

#### Descriptive analyses

Cohort characteristics, Health conditions in individuals and family health histories were reported using frequencies and percentages in the entire cohort and stratified by outcome mental disorder diagnosis in the individuals. The 20 health conditions with the highest imbalance, based on standardized differences, in their prevalence between individuals with and without mental disorders were selected for the descriptive analyses. Tetrachoric correlations were calculated among individual, parent and grandparent health conditions [52]. The condition with the lowest prevalence in pairs where correlations exceeded 0.8 was excluded from the models [53, 54]. 

#### Model development

Least Absolute Shrinkage and Selection Operator (LASSO) logistic regression was selected for predicting mental disorders due to its effectiveness in handling high-dimensional data as it reduces model complexity and prevents overfitting by shrinking coefficients of less important predictors to zero, thereby selecting only the most influential variables for inclusion in the predictive model [55, 56]. Four models were sequentially constructed for each outcome: base (demographic information and family history of the mental disorder outcome), individual (base model predictors plus 130 individual health conditions), parental (individual model predictors plus 130 parental health conditions), and grandparent (parental model predictors plus 130 grandparent health conditions; see Additional file 4). All models were fit in the training data and evaluated in the test data; 10-fold cross-validation was adopted for model evaluation. Two subgroup analyses were conducted to assess the potential for selection bias due to incomplete parental or grandparent linkages. First, we compared the performance of prediction models that included individual and parental health conditions in individuals with and without grandparent linkage. Second, we compared the performance of prediction models that included individual health conditions in individuals with and without parental linkage. These analyses allowed us to evaluate whether missing family linkages affected model performance and assess the representativeness of the study cohort.

#### Model evaluation

Model performance was evaluated using the area under the receiver operating characteristic curve (AUC), sensitivity, specificity, positive predictive value (PPV), negative predictive value (NPV) and Brier score, along with the 95% confidence intervals (95% CI). We interpreted AUC values using commonly cited thresholds: poor (0.50–0.69), acceptable (0.70–0.79), excellent (0.8–0.89) or outstanding discrimination (≥ 0.9) [57]. While these thresholds are not universally standardized, they are commonly used in clinical and epidemiological prediction studies as a general guide for interpreting model performance. Brier score is a measure of prediction error, which ranges from 0 to 1, with a lower value indicating lower error [58]. Predicted probability thresholds that produced the best model performance were selected to report final model performance measures. Predictor importance was described using odds ratios (OR). Average model performance and variable importance over the ten folds were reported. All analyses were performed using SAS^®^ version 9.4 (SAS Institute, Cary, North Carolina, United States). This study was approved by the Health Research Ethics Board of the University of Manitoba, and the Manitoba Health Information Privacy Committee (now the Provincial Health Research Privacy Committee) approved data access.

## Results

### Cohort description

**Fig. 1:**
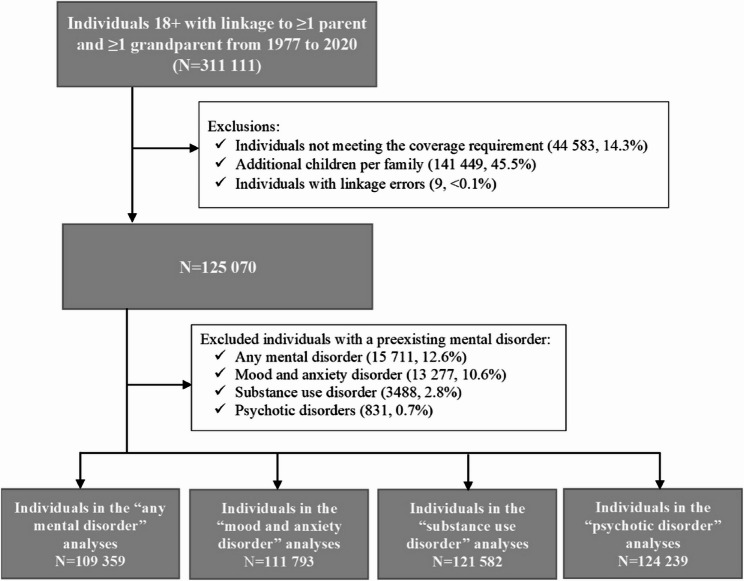
Flow diagram describing the creation of the study cohort. Note: the exclusions of preexisting mental disorders for each mental disorder outcome are independent, resulting in four cohorts for each outcome

The cohort included 125 070 individuals who met the inclusion criteria; of those 109 359 (87.4%) had no preexisting mental disorder (Fig. 1). Most of the cohort (99.1%) were linked to mothers; 68.1% were linked to fathers. All individuals were 18 years old at index date; 52.9% were males and 52.8% were urban residents. The majority of the cohort (90.9%) had a family history of a mental disorder, and a third (36.3%) had a mental disorder diagnosis recorded anytime during follow-up; mood and anxiety disorders were the most common (88.2%). Individuals with an incident mental disorder were more likely to be females, urban and lower-income neighbourhood residents (Table 1). A difference in the prevalence of individual and family histories of neurological, cardiovascular, metabolic, genitourinary, reproductive, and gastrointestinal conditions was observed between individuals with and without mental disorders.

**Table 1:** Descriptives of baseline characteristics in the study cohort and top 20 imbalanced health conditions

Characteristic	Any mental disorder diagnosis		Standardized differences	Overall
	Yes *N* = 39 651 (36.3%)	No *N* = 69 708 (63.7%)		*N* = 109 359
Sex				
Female	22 536 (56.8)	28 983 (41.6)	0.31	51 519 (47.1)
Male	17 115 (43.2)	40 725 (58.4)	0.31	57 840 (52.9)
Region of residence				
Rural	17 266 (43.5)	34 324 (49.2)	0.11	51 590 (47.2)
Urban	22 385 (56.5)	35 384 (50.8)	0.11	57 769 (52.8)
Income quintile				
Q1 (lowest)	6750 (17.0)	10 788 (15.5)	0.04	17 538 (16.0)
Q2	7371 (18.6)	12 729 (18.3)	0.01	20 100 (18.4)
Q3	7753 (19.6)	13 892 (19.9)	0.01	21 645 (19.8)
Q4	8565 (21.6)	15 335 (22.0)	0.01	23 900 (21.9)
Q5 (highest)	8858 (22.3)	16 501 (23.7)	0.03	25 359 (23.2)
Missing	354 (0.9)	463 (0.7)	0.03	817 (0.7)
Parental history of mental disorders	31 318 (79.0)	52 138 (74.8)	0.10	83 456 (76.3)
Grandparent history of mental disorders	32 601 (82.2)	59 052 (84.7)	0.07	91 653 (83.8)
Health conditions				
Grandparent dementia and other cognitive disorders	11 124 (28.1)	27 340 (39.2)	0.24	38 464 (35.2)
Individual’s menstrual disorders	11 368 (28.7)	13 743 (19.7)	0.21	25 111 (23.0)
Grandparent cataract	24 367 (61.5)	49 110 (70.5)	0.19	73 477 (67.2)
Grandparent esophageal disorders	21 579 (54.4)	44 008 (63.1)	0.18	65 587 (60.0)
Individual’s female genital disorders	7033 (17.7)	8055 (11.6)	0.18	15 088 (13.8)
Parental hypertension	20 501 (51.7)	41 946 (60.2)	0.17	62 447 (57.1)
Parental lipid metabolism disorders	16 764 (42.3)	35 330 (50.7)	0.17	52 094 (47.6)
Grandparent lipid metabolism disorders	24 731 (62.4)	48 898 (70.2)	0.17	73 629 (67.3)
Parental cataract	2321 (5.9)	7199 (10.3)	0.16	9520 (8.7)
Parental menopausal disorder	11 564 (29.2)	25 529 (36.6)	0.16	37 093 (33.9)
Parental osteoarthritis	13 564 (34.2)	29 213 (41.9)	0.16	42 777 (39.1)
Grandparent chronic kidney disease and related conditions	10 218 (25.8)	22 986 (33.0)	0.16	33 204 (30.4)
Grandparent anemia	25 052 (63.2)	49 212 (70.6)	0.16	74 264 (67.9)
Individual’s other diseases of bladder and urethra	16 263 (41.0)	23 466 (33.7)	0.15	39 729 (36.3)
Parental esophageal disorders	13 135 (33.1)	28 174 (40.4)	0.15	41 309 (37.8)
Grandparent genitourinary congenital anomalies	2769 (7.0)	7895 (11.3)	0.15	10 664 (9.8)
Grandparent other nervous system conditions	23 883 (60.2)	46 956 (67.4)	0.15	70 839 (64.8)
Parental other nervous system conditions	15 334 (38.7)	32 024 (45.9)	0.15	47 358 (43.3)
Parental hyperplasia of prostate	2515 (6.3)	7111 (10.2)	0.14	9626 (8.8)
Individual’s urinary tract infections	5898 (14.9)	7184 (10.3)	0.14	13 082 (12.0)

The 20 health conditions with the largest standardized differences in prevalence among individuals with and without a mental disorder diagnosis were reported

### Model evaluation

#### Prediction performance

**Fig. 2:**
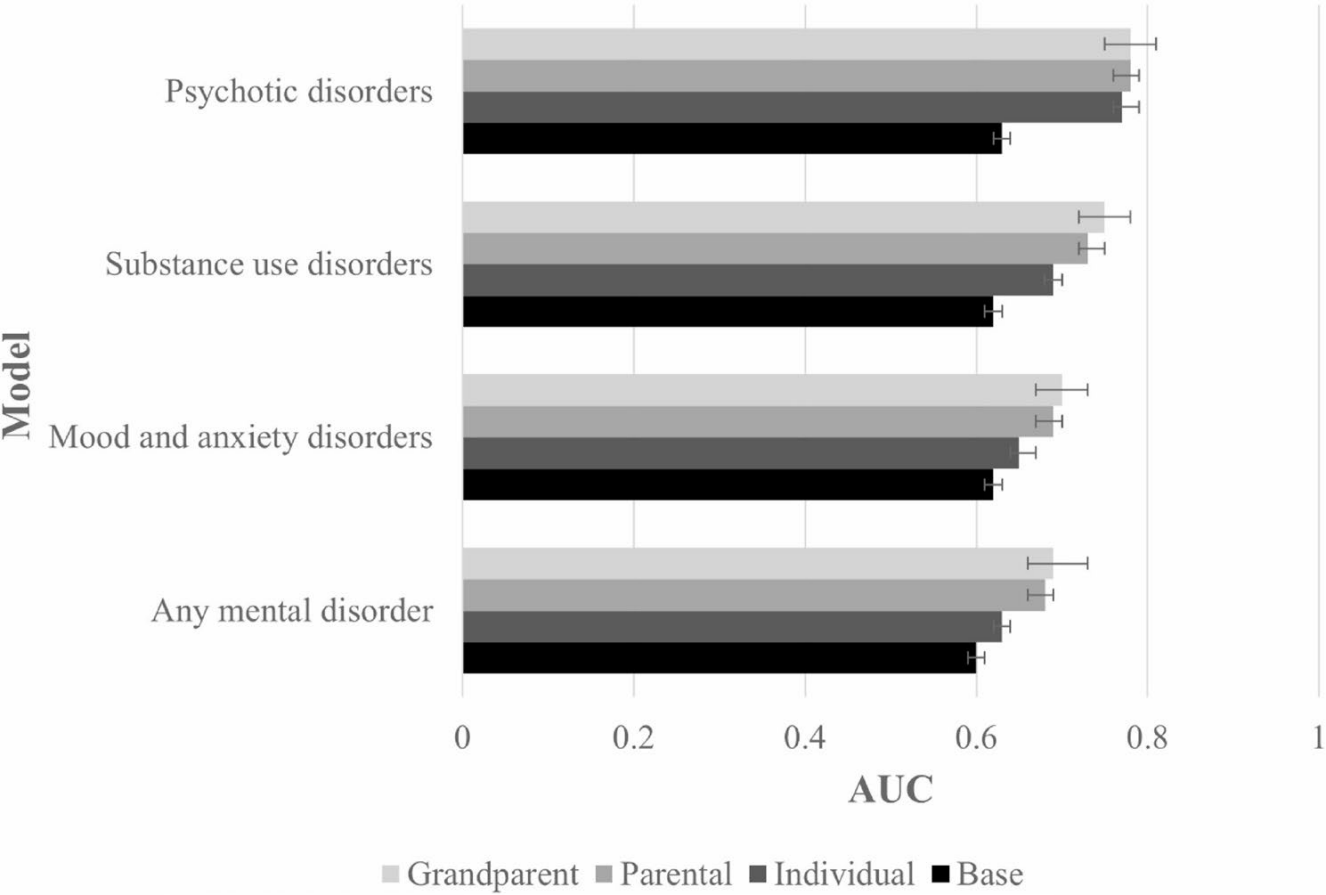
Area under the receiver operating characteristic curve (AUC) of LASSO logistic regression models predicting mental disorder risk

Table 2 lists the evaluation metrics for the developed models. For any mental disorder outcome, the base model achieved poor discrimination with AUC of 0.60 (95% CI 0.59–0.61), sensitivity of 0.57 (95% CI 0.55–0.58) and specificity of 0.58 (95% CI 0.57–0.60). Including individual and family health conditions in the models improved the predictive performance, with AUCs of 0.63 (95% CI 0.62–0.64), 0.68 (95% CI 0.67–0.69), and 0.69 (95% CI 0.68–0.70) in individual, parental and grandparent models, respectively (Fig. 2). The best performance was achieved with the grandparent model, which had a sensitivity of 0.65 (95% CI 0.64–0.67) and specificity of 0.63 (95% CI 0.62–0.64). Similar findings were observed for mood and anxiety and substance use disorder outcomes; grandparent models achieved the best predictive accuracies with acceptable discrimination: mood and anxiety AUC: 0.70 (95% CI 0.69–0.72), sensitivity: 0.66 (95% CI 0.65–0.68) and specificity: 0.64 (95% CI 0.63–0.65); substance use disorders AUC: 0.75 (95% CI 0.73–0.76), sensitivity: 0.69 (95% CI 0.67–0.72) and specificity: 0.67 (95% CI 0.66–0.68) [57]. 

**Table 2:** Summary of evaluation metrics of LASSO logistic regression models predicting mental disorder risk

Model	Model evaluation metrics (95% CI)					
	AUC	Sensitivity	Specificity	PPV	NPV	Brier score
Any mental disorder (predicted probability threshold 0.36)						
Base	0.60 (0.59–0.61)	0.57 (0.55–0.58)	0.58 (0.57–0.60)	0.44 (0.42–0.45)	0.70 (0.69–0.72)	0.22 (0.22–0.23)
Individual	0.63 (0.62–0.64)	0.60 (0.59–0.62)	0.59 (0.58–0.61)	0.46 (0.44–0.47)	0.72 (0.71–0.74)	0.22 (0.22–0.22)
Parental	0.68 (0.67–0.69)	0.64 (0.62–0.65)	0.62 (0.60–0.63)	0.49 (0.47–0.50)	0.75 (0.74–0.76)	0.21 (0.21–0.21)
Grandparent	0.69 (0.68–0.70)	0.65 (0.64–0.67)	0.63 (0.62–0.64)	0.50 (0.49–0.51)	0.76 (0.75–0.77)	0.21 (0.20–0.21)
Mood and anxiety disorders (predicted probability threshold 0.32)						
Base	0.62 (0.61–0.63)	0.60 (0.59–0.62)	0.59 (0.58–0.60)	0.41 (0.40–0.43)	0.76 (0.75–0.77)	0.21 (0.21–0.21)
Individual	0.65 (0.64–0.67)	0.63 (0.61–0.64)	0.60 (0.59–0.62)	0.43 (0.42–0.44)	0.77 (0.76–0.78)	0.20 (0.20–0.21)
Parental	0.69 (0.68–0.70)	0.65 (0.64–0.67)	0.63 (0.62–0.64)	0.46 (0.44–0.47)	0.79 (0.78–0.80)	0.20 (0.19–0.20)
Grandparent	0.70 (0.69–0.72)	0.66 (0.65–0.68)	0.64 (0.63–0.65)	0.46 (0.45–0.48)	0.80 (0.79–0.81)	0.19 (0.19–0.20)
Substance use disorders (predicted probability threshold 0.11)						
Base	0.62 (0.60–0.63)	0.58 (0.55–0.60)	0.60 (0.59–0.61)	0.15 (0.14–0.16)	0.92 (0.91–0.93)	0.10 (0.09–0.10)
Individual	0.69 (0.67–0.70)	0.61 (0.59–0.64)	0.66 (0.65–0.67)	0.18 (0.17–0.19)	0.93 (0.93–0.94)	0.09 (0.09–0.10)
Parental	0.73 (0.72–0.75)	0.67 (0.65–0.70)	0.67 (0.66–0.67)	0.20 (0.19–0.21)	0.94 (0.94–0.95)	0.09 (0.09–0.09)
Grandparent	0.75 (0.73–0.76)	0.69 (0.67–0.72)	0.67 (0.66–0.68)	0.21 (0.19–0.22)	0.95 (0.94–0.95)	0.09 (0.08–0.09)
Psychotic disorders (predicted probability threshold 0.018)						
Base	0.63 (0.60–0.67)	0.63 (0.56–0.69)	0.57 (0.57–0.58)	0.03 (0.02–0.03)	0.99 (0.98–0.99)	0.02 (0.02–0.02)
Individual	0.77 (0.74–0.80)	0.69 (0.63–0.75)	0.71 (0.70–0.72)	0.04 (0.04–0.05)	0.99 (0.99–0.99)	0.02 (0.02–0.02)
Parental	0.78 (0.75–0.81)	0.72 (0.67–0.78)	0.69 (0.68–0.70)	0.04 (0.04–0.05)	0.99 (0.99–0.99)	0.02 (0.02–0.02)
Grandparent	0.78 (0.75–0.81)	0.75 (0.69–0.80)	0.68 (0.67–0.69)	0.04 (0.04–0.05)	0.99 (0.99–0.99)	0.02 (0.02–0.02)

*Abbreviations* *LASSO* Least Absolute Shrinkage and Selection Operator, *CI* confidence interval, *AUC* area under the curve, *NPV* negative predictive value, *PPV* positive predictive value

For psychotic disorders, including individual health conditions significantly improved the predictive model performance of the base model (Table 2; Fig. 2), with an AUC of 0.77 (95% CI 0.74–0.80) compared with 0.63 (0.60–0.67), sensitivity of 0.69 (95% CI 0.56–0.69) compared with 0.63 (95% CI 0.56–0.69), and specificity of 0.71 (95% CI 0.70–0.72) compared with 0.57 (95% CI 0.57–0.58). Including parental and grandparent health conditions did not further improve discriminative performance beyond the improvement obtained from including individual health conditions alone, with the AUC remaining at 0.78 (95% CI 0.75–0.81) for both models. Brier scores ranged from 0.02 for the psychotic disorders, indicating little prediction error, to 0.21 for mood and anxiety disorders, indicating greater prediction error (Table 2). Subgroup analyses showed similar AUCs of mental disorder risk prediction models in individuals with and without grandparent linkage, as well as individuals with and without parental linkage (Additional file 8).

#### Predictor importance

**Fig. 3:**
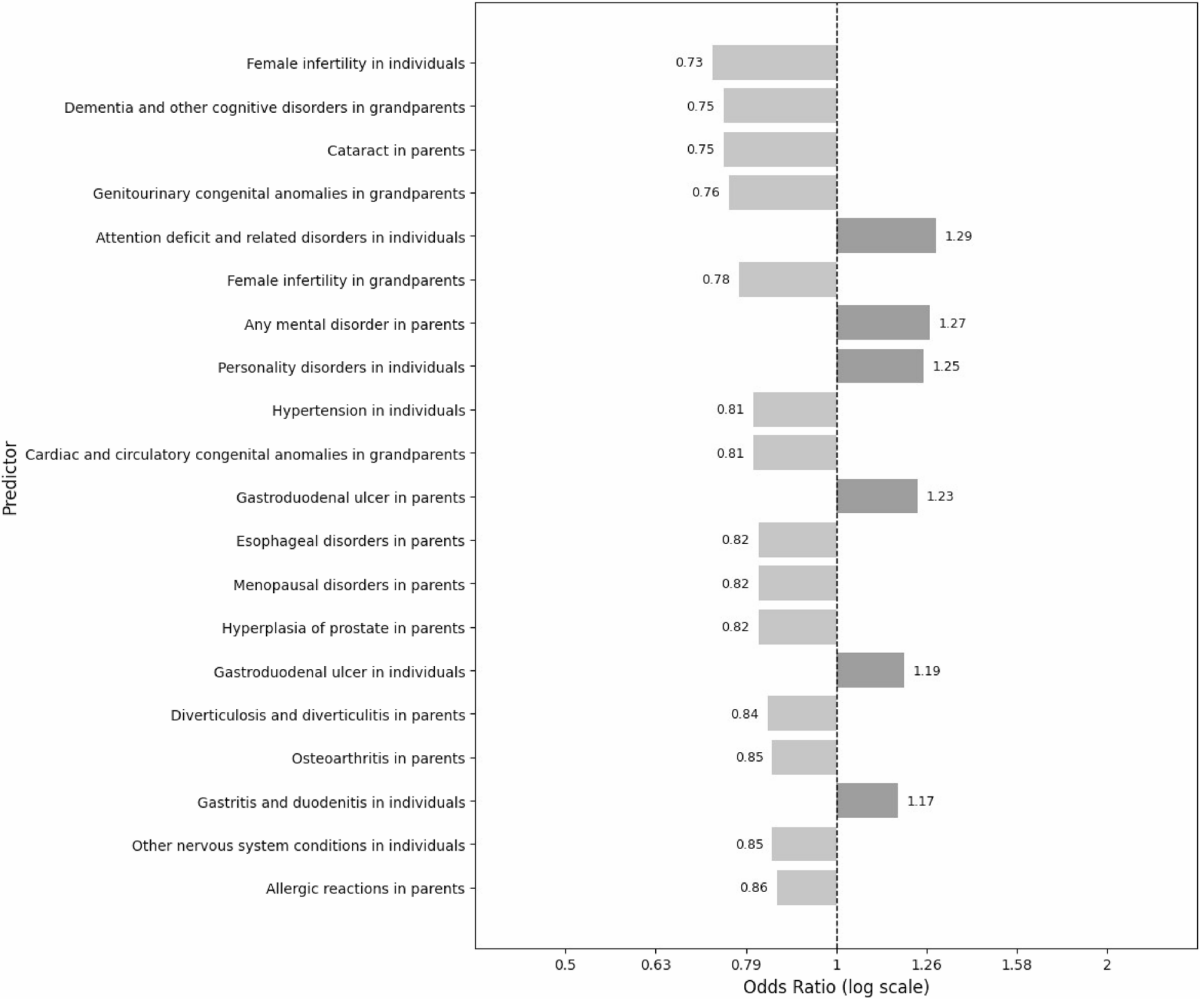
The top 20 predictors of any mental disorder among individual, parent and grandparent health conditions based on odd ratios, ordered from the most important. Note: odd ratios are penalized and many are shrunk towards zero, so we recommend caution when interpreting the magnitudes of the odd ratios from the LASSO logistic regression models

Figure 3 reports the 20 most important individual and family health condition predictors of any mental disorder, with some predictors positively and others negatively associated with the outcome. Among individual health conditions, female infertility, attention deficit and related disorders and personality disorders were the most important predictors of mental disorders. Parental cataract, mental disorders, and gastroduodenal ulcers; and grandparent dementia and other cognitive disorders, genitourinary congenital anomalies and female infertility were the most important predictors among the family health histories. Additional files 5–7 list the 20 most important predictors for the other study outcomes: mood and anxiety, substance use and psychotic disorders. The most important predictors for mood and anxiety disorders among individual health conditions were psychotic disorders, female infertility and substance use disorders; among parental health conditions were cataract, mood and anxiety disorders and esophageal disorders; and among grandparent health conditions were dementia and other cognitive disorders, genitourinary congenital anomalies and female infertility. For substance use disorders, individual psychotic, personality and adjustment disorders, parental substance use disorders, cataract and gastroduodenal ulcer, and grandparent dementia and other cognitive disorders, lipid metabolism disorders and genitourinary congenital anomalies were among the most important predictors. For psychotic disorders, individual personality, substance use and mood and anxiety disorders, parental psychotic disorders, lipid metabolism disorders and nervous system conditions, and grandparent lipid metabolism disorders, dementia and other cognitive disorders, and thyroid disorders were among the most important predictors.

## Discussion

In this population-based multigenerational study, we built prediction models of mental disorder risk with acceptable discrimination using a comprehensive list of individual and family health histories. Compared with base models that included demographic predictors and family history of the outcome, including comprehensive individual and family mental and physical health histories improved the prediction accuracies for most mental disorder outcomes. However, incorporating family health histories did not significantly improve predictive models of psychotic disorders beyond those achieved by including individual health histories. The primary aim of this study was to explore and identify potential novel multigenerational predictors of mental disorder risk using population-based administrative data, rather than to develop a clinical prediction tool. While no single health condition emerged as a strong stand-alone predictor, including a broad set of conditions across three generations collectively improved model performance.

Among existing prediction models of mental disorder risk, which primarily focused on mood and anxiety disorders, only a few have considered family health histories. Those that included family health histories focused on mental health and did not consider physical health. For example, studies included a family history of schizophrenia in predicting psychosis risk, a family history of any mental disorder in predicting depressive disorder and psychotic disorder risk, and substance and alcohol use history in predicting substance and alcohol use risk in small populations of up to 3000 individuals [39, 43–45]. Our results demonstrate that including a broad range of objectively ascertained mental and physical health histories across three generations can improve the predictive accuracy of mental disorder models in a general population cohort. This study offers a novel population-based perspective on intergenerational contributors to mental disorder risk, extending prior work that focused primarily on self-reported, single-generation histories and a narrow set of conditions. Despite comprehensive predictors, predictive accuracy was modest. This likely reflects the complex and multifactorial nature of mental disorders, which are influenced by factors not well captured in administrative data, such as childhood adversity, social support and environmental exposures. In addition, misclassification, underdiagnosis, and lack of data on disease severity may further limit prediction accuracy. However, these predictive models, despite having modest accuracy, may still be valuable for public health planning and population-level risk stratification, even if they are not yet suitable for clinical decision-making at the individual level.

In all mental disorder outcome models, histories of other mental disorders in individuals were among the most important predictors. For example, psychotic and substance use disorders in individuals were important predictors of their mood and anxiety disorder risk; psychotic, personality and adjustment disorders in individuals were important predictors of their substance use disorder risk; and personality, substance use and mood and anxiety disorders in individuals were important predictors of their psychotic disorder risk. This indicates a high prevalence of comorbidity across mental disorders, likely due to shared genetic liability, biological pathways or common environmental and social determinants [59–62]. Similarly, parental mood and anxiety, substance use and psychotic disorder histories were each important predictors of the risks of those respective outcomes. This finding demonstrates that parental mental disorder history serves as an important disorder-specific risk factor, highlighting the importance of incorporating parental history in assessing individuals’ risk of the disorders.

While mental health conditions in grandparents were not among the most important predictors of mental disorder risk, physical health conditions, such as dementia, and lipid metabolism disorders in grandparents, were found to be important predictors of mental disorder risk. Notably, several important predictors among individual and family physical health conditions, such as female infertility, lipid metabolism disorders, dementia, and cataract, had inverse associations with mental disorder risk. These apparent protective effects may be due to non-biological mechanisms, as they may reflect increased healthcare encounters related to these conditions, which provide opportunities for screening and early intervention, or unmeasured shared social and environmental factors, such as socioeconomic and family support dynamics [22, 63, 64]. In contrast, gastrointestinal conditions, such as ulcers and gastritis in individuals and parents, were identified as important predictors that increased mental disorder risk. This could be attributed to the role of systemic inflammation and chronic stress, resulting in increased mood and anxiety symptoms in parents, making individuals more vulnerable to mental health issues through genetic and environmental pathways [65–68]. These findings highlight the need for research to understand biological and social pathways linking physical and mental disorders across generations and the role of multigenerational social determinants of health.

The main study strengths are the large study population, the use of objective measures of health histories across three generations, the inclusion of a wide range of physical and mental health conditions and multiple mental disorder outcomes. However, there are a few limitations to note. Some cohort members lacked complete parent and grandparent linkages. Previous work has shown that the ability to link fathers, and therefore paternal grandparents, using FRNs has declined over time, likely suggesting an increase in non-traditional family structures and unreported paternal relationships [69]. This could result in an underestimation of the predictive contribution of family health histories; however, subgroup analyses showed similar predictive model performance in individuals with and without grandparent or parental linkage. Additionally, misclassification of study outcomes and predictors could have resulted from coding errors, misdiagnoses, or coding and diagnosis criteria changes over time, influencing predictive model accuracies. The models did not include drug dispensations, which could serve as proxies for disease severity and influence the progression or detection of mental disorders and other health conditions. Another limitation is that individuals born outside Manitoba are less likely to have linkages to their parents in administrative data, unless they moved to the province during childhood. This limits the generalizability of the findings, particularly to the immigrant population and reflects a challenge in using administrative data for multigenerational research. Finally, the data source did not include other clinical, social and environmental factors that could improve the prediction performance of mental disorder risk, such as smoking, household dysfunction and adverse childhood experiences. Future studies that combine individual and parental health histories with clinical, social and environmental risk factors in mental disorder risk prediction can produce more accurate models.

## Conclusions

Incorporating individual and family health histories improved the performance of mental disorder risk prediction, though predictive accuracies remained modest. Future research should aim to integrate comprehensive clinical, social, and environmental data in mental disorder risk prediction, enhancing early risk identification and enabling targeted preventive interventions.

## Supplementary Information

Additional file 1: A diagram showing the timeline of ICD versions used to report diagnoses in Hospital Abstracts and Medical Claims.

Additional file 2: A diagram of the study timeline.

Additional file 3: A table listing the International Classification of Diseases (ICD) codes used to identify the study outcomes.

Additional file 4: A list of the developed prediction models and the predictors included in each model.

Additional file 5: The top 20 predictors of mood or anxiety disorders among individual, parent and grandparent health conditions based on odd ratios, ordered from the most important.

Additional file 6: The top 20 predictors of substance use disorders among individual, parent and grandparent health conditions based on odd ratios, ordered from the most important.

Additional file 7: The top 20 predictors of psychotic disorders among individual, parent and grandparent health conditions based on odd ratios, ordered from the most important.

Additional file 8: Area under the receiver operating characteristic curve (AUC) and 95% confidence intervals of LASSO logistic regression models predicting mental disorder risk in subgroup analyses.

## Data Availability

Data used in this article were derived from administrative health data as a secondary use. The data were provided under specific data sharing agreements only for approved use at the Manitoba Centre for Health Policy (MCHP). The original source data are not owned by the researchers or MCHP and as such, cannot be provided to a public repository. The original data source and approval for use have been noted in the acknowledgments of the article. Where necessary, source data specific to this article or project may be reviewed at MCHP with the consent of the original data providers, along with the required privacy and ethical review bodies.

## Abbreviations

FRN: Family Identification Number
ICD: The International Classification of Diseases
ICD-8: The International Classification of Diseases, the eighth revisions
ICD-9-CM: The International Classification of Diseases, the ninth revision with clinical modifications
ICD-10-CA: The International Classification of Diseases, the tenth revision with Canadian adaptations
Q: Quintile
LASSO: Least Absolute Shrinkage and Selection Operator
AUC: The area under the receiver operating characteristic curve
PPV: Positive predictive value
NPV: Negative predictive value
CI: Confidence interval
OR: Odds ratio
