# Supplementary material for: Leveraging multigenerational health data to enhance mental disorder risk prediction: a population-based cohort study
Source: BMC Psychiatry. 2025 Sep 25;25:862. doi: 10.1186/s12888-025-07323-z (PMC12465338; doi:10.1186/s12888-025-07323-z)
Supplement: Supplementary file 1 — Additional file 1: A diagram showing the timeline of ICD versions used to report diagnoses in Hospital Abstracts and Medical Claims. [file 12888_2025_7323_MOESM1_ESM.docx]

**
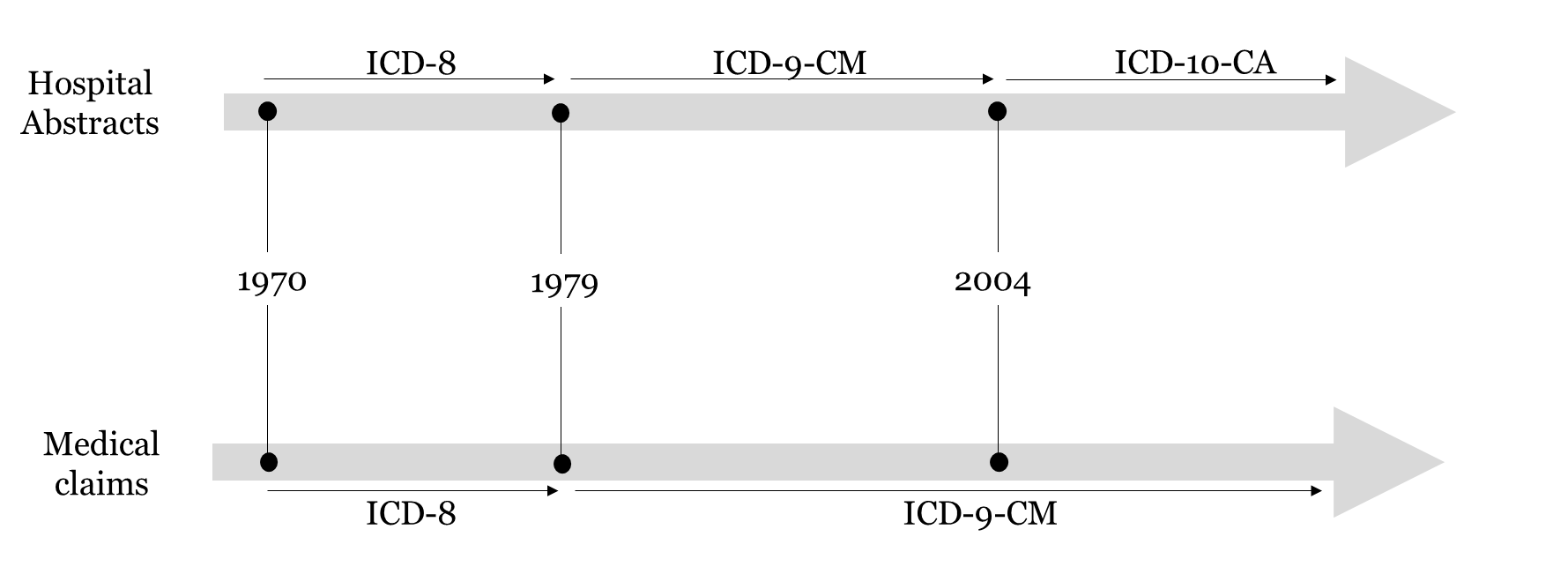
**

Additional file 1. A diagram showing the timeline of ICD versions used to report diagnoses in Hospital Abstracts and Medical Claims.
