# Supplementary material for: Leveraging multigenerational health data to enhance mental disorder risk prediction: a population-based cohort study
Source: BMC Psychiatry. 2025 Sep 25;25:862. doi: 10.1186/s12888-025-07323-z (PMC12465338; doi:10.1186/s12888-025-07323-z)
Supplement: Supplementary file 2 — Additional file 2: A diagram of the study timeline. [file 12888_2025_7323_MOESM2_ESM.docx]

**
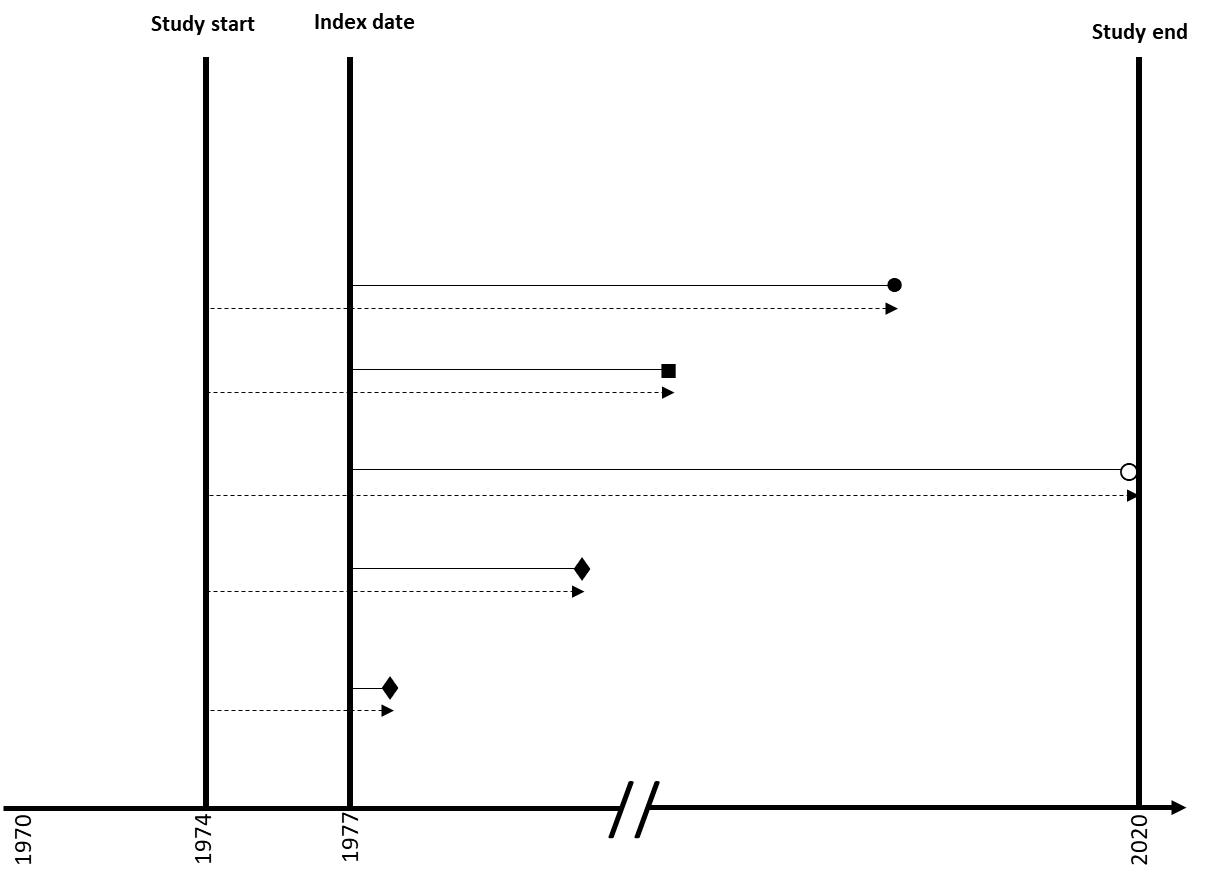
**

Additional file 2. A diagram of the study timeline. The solid line ( ) represents follow-up period (outcome ascertainment window), the dashed arrow ( ) represents predictor ascertainment window, ⚫ indicates migration out of the province, ◼ indicates death, ⭘ indicates end of study period, ⧫ indicates the outcome (mental disorder) has occurred. Note: index date was the year 1977 or the year an individual turned 18, whichever occurred later.
