# Supplementary material for: Leveraging multigenerational health data to enhance mental disorder risk prediction: a population-based cohort study
Source: BMC Psychiatry. 2025 Sep 25;25:862. doi: 10.1186/s12888-025-07323-z (PMC12465338; doi:10.1186/s12888-025-07323-z)
Supplement: Supplementary file 3 — Additional file 3: A table listing the International Classification of Diseases (ICD) codes used to identify the study outcomes. [file 12888_2025_7323_MOESM3_ESM.docx]

Additional file 3. A table listing the International Classification of Diseases (ICD) codes used to identify the study outcomes.

| **Outcome** | **ICD-8** | **ICD-9-CM** | **ICD-10-CA** |
| --- | --- | --- | --- |
| Mood and anxiety disorders | 296, 300, 307 | 296, 311, 309, 300 | F30-F34, F38, F40, F41, F41.1, F41.2, F41.3, F41.8, F41.9, F42, F43, F53 |
| Substance use disorders | 291, 294, 303, 304 | 291, 292, 303- 305 | F10-19, F55, Z50.2, Z50.3 |
| Psychotic disorders | 295, 297, 298, 299 | 295, 297, 298 | F11.5, F12.5, F13.5, F14.5, F15.5, F16.5, F17.5, F18.5, F19.5, F20-F25, F28, F29 |

ICD-8: the international classification of diseases - eighth revision; ICD-9-CM: the international classification of diseases - ninth revision with clinical modifications; ICD-10-CA: the international classification of diseases- tenth revision with Canadian adaptations (ICD-10-CA)
