# Supplementary material for: Leveraging multigenerational health data to enhance mental disorder risk prediction: a population-based cohort study
Source: BMC Psychiatry. 2025 Sep 25;25:862. doi: 10.1186/s12888-025-07323-z (PMC12465338; doi:10.1186/s12888-025-07323-z)
Supplement: Supplementary file 4 — Additional file 4: A list of the developed prediction models and the predictors included in each model. [file 12888_2025_7323_MOESM4_ESM.docx]

Additional file 4. A list of the developed prediction models and the predictors included in each model.

| **Model** | **Predictors** |
| --- | --- |
| Base | - Sex (male or female) - Income quintile (Q1-Q5) - Region of residence (urban or rural) - Family history of the outcome measure in a parent - Family history of the outcome measure in a grandparent |
| Individual | - All base model predictors - 130 health conditions for the individual (excluding the outcome) |
| Parental | - All base model predictors - 130 health conditions for the individual (excluding the outcome) - 130 health conditions for either parent |
| Grandparent | - All base model predictors - 130 health conditions for the individual (excluding the outcome) - 130 health conditions for either parent - 130 health conditions for any grandparent |

Note: case definitions for the 130 health conditions are based on the Clinical Classification Software and reported in Hamad AF et al. Int J Popul Data Sci. 2021. Some predictors were excluded due to collinearity or inclusion in the base model.
