# Supplementary material for: Leveraging multigenerational health data to enhance mental disorder risk prediction: a population-based cohort study
Source: BMC Psychiatry. 2025 Sep 25;25:862. doi: 10.1186/s12888-025-07323-z (PMC12465338; doi:10.1186/s12888-025-07323-z)

Additional file 6. The top 20 predictors of substance use disorders among individual, parent and grandparent health conditions based on odd ratios, ordered from the most important.


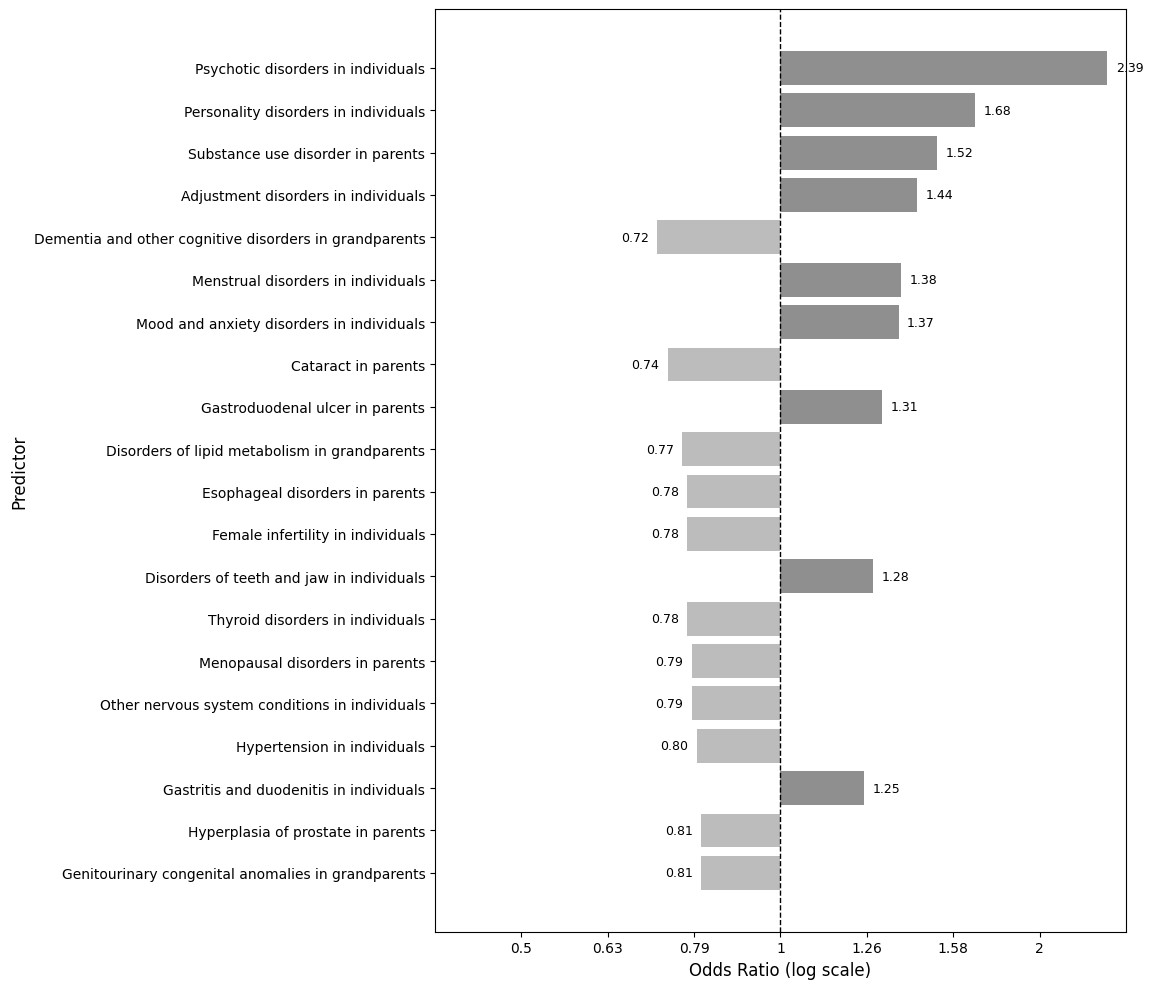

Supplement: Supplementary file 6 — Additional file 6: The top 20 predictors of substance use disorders among individual, parent and grandparent health conditions based on odd ratios, ordered from the most important. [file 12888_2025_7323_MOESM6_ESM.docx]
