# Supplementary material for: Leveraging multigenerational health data to enhance mental disorder risk prediction: a population-based cohort study
Source: BMC Psychiatry. 2025 Sep 25;25:862. doi: 10.1186/s12888-025-07323-z (PMC12465338; doi:10.1186/s12888-025-07323-z)
Supplement: Supplementary file 8 — Additional file 8: Area under the receiver operating characteristic curve (AUC) and 95% confidence intervals of LASSO logistic regression models predicting mental disorder risk in subgroup analyses. [file 12888_2025_7323_MOESM8_ESM.docx]

Additional file 8. Area under the receiver operating characteristic curve (AUC) and 95% confidence intervals of LASSO logistic regression models predicting mental disorder risk in subgroup analyses
